# Attitudes of orthodontists and laypersons towards tooth extractions and additional anchorage devices

**DOI:** 10.1186/s40510-017-0174-0

**Published:** 2017-07-24

**Authors:** Chidsanu Changsiripun, Petchpailin Phusantisampan

**Affiliations:** Department of Orthodontics, Faculty of Dentistry, Chulalongkorn University, Henri-Dunant Road, Wangmai, Patumwan, Bangkok, 10330 Thailand

**Keywords:** Extraction, Tooth condition, Anchorage devices, Orthodontists, Laypersons

## Abstract

**Background:**

This study investigated the attitudes of orthodontists and laypersons towards the choice of extracting second premolars, rather than first premolars, based on tooth condition and the use of additional anchorage devices.

**Methods:**

Questionnaires were sent to two groups: 324 orthodontists who were members of the Thai Association of Orthodontists, and 100 randomly selected Thai laypersons aged above 20 years and who were unrelated to the field of dentistry. Descriptive and chi-square statistics were used to analyze the data.

**Results:**

Questionnaires were returned by 142 orthodontists (43.8%) and completed by 100 laypersons. The larger the size of the caries lesion in the maxillary second premolar was found, the more orthodontists and laypersons both chose to extract a carious maxillary second premolar instead of a healthy maxillary first premolar. For orthodontists, the use of mini-implant anchorage was significantly related to their extraction decision. Orthodontists who were familiar with mini-implants usage would choose to extract the second premolar at a lower size of extent of caries. Besides, when larger sizes of caries lesions in maxillary second premolars were considered, laypersons tended to have greater acceptance of the use of additional anchorage devices in order to keep the healthy maxillary first premolar.

**Conclusions:**

In this study, tooth condition and the use of anchorage devices are currently the main considerations by both orthodontists and laypersons when selecting the teeth to be extracted for orthodontic treatment.

## Background

One of the most common dental problems bringing patients to see an orthodontist is anterior crowding and protrusion [1]. One of the treatment options to create space for solving this tooth size-arch length discrepancy is tooth extraction, which allows the remaining teeth to be moved into perfect alignment. Although the tooth misalignment problem occurs within the anterior esthetic zone, these anterior teeth should not be removed because of their specific shapes and esthetic impact. Thus, orthodontists typically choose to extract the first or second premolar because of their lower impact on esthetics and masticatory function compared to anterior teeth and molars. Being close to the problem area, the first premolar is the first choice for removal, compared with the second premolar, because it is then simpler to close the space created. In addition, keeping the second premolar helps control the anchorage required to relieve the anterior crowding. Therefore, most orthodontists would choose to remove the first premolar to correct anterior protrusion or crowding and to meet two of the goals of orthodontic treatment, i.e., minimizing treatment time and minimizing the distance the teeth must be moved [2].

Mini-implants play an important role in modern orthodontic treatment planning as they can be the absolute anchorage control [3–5]. Consequently, extracting the closest tooth to the problem area may no longer be the best choice for all moderate to severe crowding patients. Instead, the concern seemed to shift to the long-term prognosis for the tooth when selecting extraction sites in orthodontic treatment. However, up to date, we still cannot find enough studies which support this assumption.

Orthodontic treatment is considered to be a long continuous process, compared with other dental treatments, and patient compliance is essential for treatment success. From personal experiences, it has been found that patients who understand their condition and accept the proposed treatment plan are more compliant. Therefore, studying the attitude of laypersons regarding their preference of which tooth to remove might be meaningful for orthodontic treatment planning. However, currently, there are no data regarding patients’ attitudes about this choice.

Previous investigations on tooth extraction for orthodontic reasons evaluated whether first or second premolar extraction decreased facial dimension [6], altered dimensional changes measured from cephalometric analysis [7], affected the soft tissue of upper lip areas [8], or allowed third molar eruption [9]. However, there are no studies concerning the relative condition of the first and second premolars when deciding which one to extract. It was reported that the second premolars are more vulnerable to caries attack than the first premolars with a ratio of 1.6:1 [10]. Thus, the present study was undertaken to answer the question as to how severe dental caries in maxillary second premolar would make orthodontists and laypersons choose to extract the maxillary second premolar, instead of a healthy maxillary first premolar, in a Class I Angle relationship with anterior crowding or protrusion. The underlying assumption was that the case had been analyzed and it had been decided to extract four bicuspids with the need for maximum anchorage in the upper arch.

## Methods

### Questionnaire

A modified version of the Mount and Hume [11] Caries Classification System was used in our self-administered questionnaires as a measure of the size of the caries lesion. This questionnaire divided the size of lesions into seven levels, from 0 to 6 (Table 1). We developed our own questionnaires based on this classification, which comprised two main parts for both orthodontists and laypersons to obtain:

**Table 1:** Classification of the severity of caries progression used in the present study (modified from Mount and Hume [11])

Classification of dental caries	Meaning
Size 0	Healthy tooth.
Size 1	Only demineralization but no cavitation. Remineralization treatment can stop the process of developing disease.
Size 2	Minimal involvement of dentine just beyond treatment by remineralization alone.
Size 3	Moderate involvement of dentine. The remaining tooth structure is sufficiently strong to support the restoration and not likely to fail under normal occlusal load.
Size 4	The cavity is enlarged beyond moderate. The remaining tooth structure is weakened to the extent that cusps or incisal edges are split or are likely to fail if left exposed to occlusal or incisal load.
Size 5	Extensive caries with bulk loss of tooth structure has already occurred.
Size 6	Exposed pulp caries with extensive loss of enamel and dentine. Root canal treatment followed by crown restoration is necessary in order to maintain the tooth.

Part I: general information of respondent such as gender, age, educational degree, and orthodontic work experience.

Part II: attitudes concerning removal of the maxillary second premolar, rather than the maxillary first premolar, according to the scenario described above and attitudes towards the use of anchorage devices.

### Sample accrual

The study population was divided into two groups. The first group was composed of all 324 active members of the Thai Association of Orthodontists. The second group included 100 Thai laypersons above 20 years old, who would like to receive orthodontic treatment at the orthodontic clinic, Faculty of Dentistry, Chulalongkorn University, and were not related to the field of dentistry. An identical patient education video was shown to all potential orthodontic patients before they filled a questionnaire.

### Data analysis

Statistical analyses were performed by descriptive and chi-square analysis with SPSS software version 17.00 (SPSS Inc., Chicago, IL, USA). Statistical significance was determined at *P* < 0.05.

## Results

Questionnaires were returned by 142 orthodontists (a response rate of approximately 43.8%) and a hundred laypersons were randomly surveyed individually. Some returned questionnaires were incomplete, hence the discrepancy in response numbers between individual items.

### Demographic characteristics

The orthodontists’ demographic details are displayed in Table 2. The respondents were predominantly female (68.3%), almost half were 31–40 years old (46.5%) and approximately one third (31.7%) had up to 5 years’ experience as an orthodontist.

**Table 2:** Orthodontists’ demographic characteristics

	Number	Percentage
Orthodontists (*n* = 142)		
Male	45	31.7
Female	97	68.3
Age (years, *n* = 142)		
≤30	11	7.7
31–40	66	46.5
41–50	47	33.1
51–60	15	10.6
≥61	3	2.1
Work experience as orthodontists (years, *n* = 138)		
≤5	45	31.7
6–10	36	25.4
11–15	23	16.2
16–20	14	9.9
21–25	11	7.7
≥25	9	6.3

The demographics of laypersons are shown in Table 3. More than half of the respondents were female (61%), approximately two thirds were less than 30 years old (76%), and the majority (80%) had a bachelor’s degree as their highest level of education.

**Table 3:** Laypersons’ demographic characteristics (*n* = 100)

	Number	Percentage
Laypersons		
Male	39	39
Female	61	61
Age (years)		
≤30	76	76
31–40	10	10
41–50	8	8
51–60	5	5
≥61	1	1
Education level		
Primary school	3	3
Secondary school	6	6
Bachelor’s degree	80	80
Master’s degree	11	11

### Attitudes towards caries extent

**Fig. 1:**
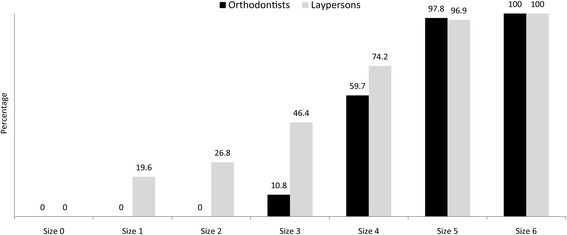
Percentage of orthodontists and laypersons who chose to remove the maxillary second premolar instead of a healthy maxillary first premolar at each size of the caries lesion in the second premolar

Regarding the scenario in the questionnaire, there were three orthodontists and three laypersons who never choose to extract the maxillary second premolar instead of the maxillary first premolar, no matter what size of the caries lesion in the second premolar. The responses of the remaining respondents to items inquiring about tooth condition are found in Fig. 1. It was found that the larger the size of the caries lesion in the maxillary second premolar, the more both orthodontists and laypersons chose to extract the maxillary second premolar rather than the maxillary first premolar. The greatest percentage of respondents in both groups, orthodontists and laypersons at 48.9 and 27.8%, respectively, chose the fourth size of caries lesion as the minimum to confirm removal of the maxillary second premolar rather than the maxillary first premolar. The lowest minimum lesion size chosen by orthodontists was 3 (10.8%), while that of laypersons was 1 (19.6%).

### Attitudes towards factors that influence an extraction decision

**Fig. 2:**
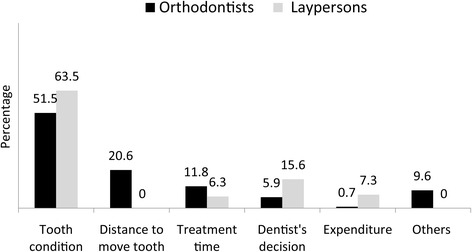
Percentage of orthodontists’ and laypersons’ attitudes towards factors that influence an extraction decision

Responses to the items enquiring about the factors influencing the respondents’ extraction decision are given in Fig. 2. More than half of the respondents in both groups (orthodontists, 51.5%; laypersons, 63.5%) agreed that tooth condition was the main factor in making the decision to remove the second premolar rather than the first premolar. The second most important factor for orthodontists was the total distance to move the anterior teeth (20.6%). Although space closure was not important to laypersons, the orthodontist’s opinion on which tooth to remove had a greater influence (15.6%). Expense was considered as the third most significant factor in laypersons’ decisions (7.3%), while only 0.7% of orthodontists took this into account.

### Attitudes towards treatment plan discussion

Almost all orthodontists agreed with discussing the treatment plan with patients, including which tooth to remove (94.2%), while 5.1% finalized the treatment plan themselves. 0.7% of responses were excluded as the treatment plan was not discussed with the patient. For laypersons, three quarters of patients agreed that it was necessary to discuss the treatment plan with the orthodontist, whereas some patients wanted to know about the treatment plan; however, the final decision depended on the orthodontist’s opinion (25%).

### Orthodontists’ attitudes towards mini-implant usage and correlation

More than half of the orthodontists’ based their decision to extract the maxillary first or second premolar on the anchorage situation (65.5%) while the remainder said it had no effect.

Responses to items enquiring about orthodontists’ usage of mini-implant anchorage are presented in Table 4. Almost half of the respondents (45.3%) always placed mini-implants themselves; one third sometimes did (31.7%), while slightly less than one fifth (18.7%) had never placed a mini-implant. Less than 5% had never used a mini-implant as the absolute anchorage.

**Table 4:** Number and percentage of orthodontists’ familiarity with the use of mini-implant anchorage (MIA) (*n* = 139)

	Number	Percentage
Orthodontists		
Always place MIA themselves	63	45.3
Sometimes place MIA themselves	44	31.7
Never placed MIA themselves	26	18.7
Never used MIA	6	4.3

Chi-square analysis indicated that there was no significant association between orthodontists’ ages and choice of lesion size indicating extraction or between their working experience and such choice. However, the lesion size was significantly related to the orthodontists’ familiarity with the use of mini-implant anchorage (MIA) (*P* = 0.04, gamma = −0.3). Thus, orthodontists who used mini-implants would choose to extract the maxillary second premolar at a smaller lesion size, compared with those who were less familiar.

### Laypersons’ attitudes towards the use of anchorage devices

**Fig. 3:**
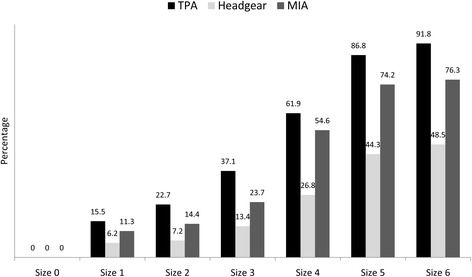
Percentage of laypersons who agreed with wearing additional anchorage devices, transpalatal arch (TPA), headgear, or mini-implant anchorage (MIA), at each size of the caries lesion in the maxillary second premolar

Responses to items enquiring about the use of different anchorage devices chosen by laypersons are shown in Fig. 3. If the maxillary second premolar was extracted rather than the maxillary first premolar, additional anchorage devices, such as a transpalatal arch (TPA), headgear, or mini-implant, need to be installed. Patients were asked to decide if they still wanted to remove the maxillary second premolar when aware of this treatment requirement. When they chose to keep the healthy maxillary first premolar, all the laypersons agreed to wear the additional devices at every size of caries lesion on the maxillary second premolar. The larger the lesion, the more laypersons agreed to wear additional devices. A TPA was the most acceptable, followed by a mini-implant, and the least popular choice was headgear at every caries lesion size.

## Discussion

Most of the chief complaints which prompt the patient to seek orthodontic treatment in any populations are either incisor protrusion or crowding [1, 12]. The presence of these clinical problems, even with a Class I molar relationship, had influenced the extraction sequence decision, and the choice of particular extraction sequences seems to have been based largely on clinical opinion [13, 14]. There have been a number of previous studies demonstrating that premolars are the most commonly extracted teeth for orthodontic purposes due to their location between anterior and posterior segments [15, 16]. When comparing first and second premolars, the first premolars are more often extracted because of their position, being located nearer to the problem site. Therefore, it is easier for anchorage control in solving the patient’s chief complaint [17]. On the other hand, when second premolars are extracted, the posterior teeth could be expected to move more forward than after a first premolar extraction, leaving inadequate remaining space for the relief of crowding and the retraction of anterior teeth [18]. This is the reason why, in the past, orthodontists almost always chose to extract the first premolars and keep the second premolars, even though the second premolars might be in far worse condition than the first premolar.

However, this was not found to be the case in the present study. We found that most orthodontists and laypersons set tooth condition as the most important factor above others: for example, space closure, treatment time, or expense, when deciding which tooth to remove. Our study demonstrated that a number of orthodontists and laypersons choosing maxillary second premolar removal instead of healthy maxillary first premolar removal increased for larger lesion sizes. That might be because most laypersons who participate in this study are well educated; 80% of them having a bachelor’s degree. They prefer to keep a healthy tooth rather than a carious tooth, even though they are informed of the requirement for the additional anchorage device. Otherwise, anterior tooth retraction or alignment of the teeth might not be optimal. In part of the orthodontists’ opinion, we found that their decision was significantly related to the familiarity with the use of MIA. Orthodontists who typically placed mini-implants themselves were likely to decide to remove the second premolar with a smaller lesion compared with those who were not familiar with mini-implant usage. This finding supported the idea of MIA causing a paradigm shift in the orthodontic world by not only making an unpredictable movement possible, such as retraction of the whole maxillary dentition in Class II division 1 malocclusions to achieve a Class I canine and molar relationship without extraction [19], intrusion of the entire maxillary dentition to correct gummy smile [20], and intrusion of the upper posterior region to correct anterior open bite [21], but also its impact on orthodontists’ decision towards extraction choice.

It is well known that closure of the premolar extraction sites occurs by retraction of anterior segments, mesial movement of posterior segments, or both. Maximum anchorage is indicated to prevent mesial movement of the posterior segments. One cephalometric study has shown that greater mean maxillary incisor retraction was found in the maxillary first premolar extraction group than in the maxillary second premolar group [7]. Therefore, patients also need to consider the additional anchorage requirement when choosing to remove the maxillary second premolar, in order to use the extraction space in a similar way to that when extracting the maxillary first premolar. In the past, headgear has been used as a standard maximum anchorage system. However, it is almost always rejected by patients because of social and esthetic concerns [22]. The present study also showed that the larger the size of the caries lesion, the higher the percentage of laypersons who accepted wearing an anchorage device, including headgear. This part of our result revealed the preference of laypersons in the twenty-first century towards the type of additional anchorage devices. Although a TPA was found to be the most popular choice, unfortunately, it was reported to be associated with anchorage loss during retraction of maxillary anterior teeth [23]. MIA, which was as effective as headgear with the non-compliance approach [24], is preferred by patients to the alternative approaches available.

To our knowledge, the present work was the first study investigating attitudes of laypersons towards their decision of tooth extraction. Nowadays, there is a growing awareness of conflict between orthodontists and patients [25]. We believe that a greater communication before starting the treatment is needed which will lead to improved relationships and to a lessening of misunderstanding. Our data supported this assumption by showing that both groups of respondents agreed that it is necessary to discuss the treatment plan together, particularly concerning tooth removal. Therefore, our results are not only helpful in the process of treatment planning between orthodontists and orthodontic patients but also could be useful for general practitioners by preventing unnecessary treatment on a severely carious second premolar if the patient intends to receive orthodontic treatment in the near future.

Nevertheless, some limitations in this study should be noted. First, the response rate from orthodontists was quite low (43.8%), although the number was almost similar to other studies using the same method in the same population [26]. In the matter of gender, the predominantly female sample of orthodontists (68.3%) could be representative of the true population (64.8% female) [26]. Second, the data acquired in this study towards extraction decision was based on one particular situation, which was to decide between maxillary first or second premolar extraction in a Class I Angle classification with anterior crowding or protrusion with the need of maximum anchorage in the upper arch. Our data showed that most of orthodontists’ extraction decision (65.5%) was influenced by how to manage the anchorage situation: maximum, moderate, or minimum. Thus, we decided to create the questionnaire by focusing only on a maximum anchorage situation for the reasons of eliminating this confounding factor and reducing the complications. Different results might also be found if it was the situation in the lower arch, as every orthodontist knows the differences in anchorage control between in the maxilla and the mandible. Therefore, this set of data should be applied with caution, and further study is required with the series of questionnaire including several types of anchorage in both arches.

## Conclusions

Tooth condition and anchorage devices are currently the main considerations when selecting which tooth to extract in orthodontic treatment for both orthodontists and laypersons in the present study.
